# Investigating the Thermal Properties of Structural Partitions Produced Using Additive Technology (3D Printing) from Biodegradable Materials for Use in Construction

**DOI:** 10.3390/ma18184379

**Published:** 2025-09-19

**Authors:** Beata Anwajler, Arkadiusz Wieleżew, Krystian Grabowski, Tullio de Rubeis, Dario Ambrosini, Ewa Zdybel, Ewa Tomaszewska-Ciosk

**Affiliations:** 1 Department of Energy Conversion Engineering, Faculty of Mechanical and Power Engineering, Wroclaw University of Science and Technology, 27 Wybrzeze Wyspianskiego Street, 50-370 Wroclaw, Poland; arkadiuszwielezew@gmail.com; 2 Institute of Psychology, University of Wroclaw, 1 J. W. Dawida St., 50-527 Wroclaw, Poland; 329860@uwr.edu.pl; 3 Department of Civil, Construction-Architectural and Environmental Engineering, University of L’Aquila, Piazzale Pontieri 1, Monteluco di Roio, 67100 L’Aquila, Italy; tullio.derubeis@univaq.it; 4 Department of Industrial and Information Engineering and Economics, University of L’Aquila, Piazzale Pontieri 1, Monteluco di Roio, 67100 L’Aquila, Italy; dario.ambrosini@univaq.it; 5 Department of Food Storage and Technology, Wroclaw University of Environmental and Life Sciences, 25 Norwida Street, 50-375 Wroclaw, Poland; ewa.zdybel@upwr.edu.pl (E.Z.); ewa.tomaszewska-ciosk@upwr.edu.pl (E.T.-C.)

**Keywords:** physical properties, structural partitions, additive technology, 3D printing, biodegradable materials

## Abstract

Advancements in material technologies and increasingly stringent thermal insulation requirements are driving the search for innovative solutions to serve as an alternative to traditional insulating materials. Using 3D printing techniques to produce thermal insulation opens up new possibilities for creating structures, geometries, and shapes from a variety of raw materials, ranging from synthetic polymers to biodegradable composites. This study aimed to develop a modern thermal insulation barrier with a comparable thermal conductivity to conventional materials to enhance the energy efficiency of buildings. Cellular materials based on the Kelvin cell were fabricated using additive manufacturing via 3D SLS printing from a composite consisting of a biodegradable material (TPS) and a recyclable polymer (PA12). The printed cellular structural partitions were tested for their thermal insulation properties, including thermal conductivity coefficient, thermal transmittance (U-value), and thermal resistance. The best thermal insulation performance was demonstrated by a double-layer partition made from TPS + PA12 at a mass ratio of 5:5 and with a thickness of 60 mm. This sample achieved a thermal conductivity of λ = 0.026 W/(m·K), a thermal resistance of R = 2.4 (m^2^·K)/W, and a thermal transmittance of U = 0.42 W/(m^2^·K). Cellular partition variants with the most favorable properties were incorporated into building thermal balance software and an energy simulation was conducted for a single-family house using prototype insulating materials. This enabled an assessment of their energy efficiency and cost-effectiveness.

## 1. Introduction

Global warming and associated climate change are among the greatest challenges facing the modern world. The construction industry relies heavily on raw materials and energy-intensive technologies, and therefore plays a significant role in increasing environmental pressures through the depletion of non-renewable resources and the intensification of CO_2_ emissions [1]. According to the latest GlobalABC/UNEP reports, the building sector accounts for around 34% of global energy demand and approximately 34% of energy- and process-related CO_2_ emissions [2,3]. Meanwhile, the International Energy Agency (IEA) predicts that demand for cooling will triple by 2050. Space cooling is currently one of the fastest-growing components of energy demand, particularly in warm regions of Africa, South America, and Asia. The IEA estimates that around 2 billion air-conditioning units are already in operation worldwide, significantly contributing to peak electricity demand [4]. The scale of this challenge is further amplified by the rapid urbanization of the Global South. In this context, affordable, locally 3D-printed, and bio-based wall insulation materials could be a key solution for reducing peak energy demand and improving thermal comfort in hot climates [3,4]. Bio-derived solutions in the form of innovative, low-emission, and passive insulation systems combining materials with optimized geometries are particularly important outside the European Union—especially in regions where high energy costs and limited access to cooling hinder development.

The foundation of any building renovation aimed at improving energy efficiency is comprehensive thermal modernization [5,6]. This involves reducing heat loss through external walls, among other measures [7,8]. The most effective way to improve a building’s thermal performance is to insulate its external walls using conventional materials such as mineral wool or polystyrene [9]. Technological advances in materials, combined with increasingly stringent thermal insulation requirements, are prompting the development of innovative alternative insulation solutions [10,11,12,13,14,15,16]. Table 1, Table 2, Table 3 and Table 4 present selected examples of the use of biomaterials in thermal insulation structures.

Using 3D printing techniques to produce thermal insulation creates new opportunities for designing structures, shapes, and geometries from different raw materials, such as plastics and biodegradable composites [65]. Three-dimensional printing allows for the localized processing of regional biomass materials such as straw, sawdust, plant husks, and cork. This promotes supply chain independence and reduces insulation costs. Additive manufacturing is currently receiving a lot of attention due to its ability to produce customized objects. It has numerous potential applications, including rapid prototyping, biomedicine (e.g., scaffolds for bone tissue engineering), and the textile and aerospace industries. It enables large-scale customization [66].

The growing industry demand for advanced insulation technologies has prompted research into using 3D printing methods to create high-performance thermal insulation materials [22,65,67,68,69,70,71,72]. Additively manufactured insulation materials are characterized by their unique and complex geometries [65]. Researchers have focused on cellular geometries with regular and irregular internal structures, including symmetric patterns, Schwarz D surfaces, Schwarz P surfaces, grids, Hilbert curves, linear and rectangular arrangements, hexagons, honeycombs, and spirals [73,74,75,76]. These topologies offer diverse thermal and mechanical properties, enabling engineers and designers to create customized solutions by balancing thermal insulation, mechanical strength, and production costs. Due to their insulating performance and thin layers, these materials can replace conventional insulation methods in the construction sector. The thermal conductivity of these materials depends on the structure type and the feedstock used for printing. This study aims to develop innovative thermal insulation materials using additive manufacturing technology with a thermal conductivity similar to conventional materials. These materials could improve building energy efficiency. Using recyclable and biodegradable feedstocks would align with the principles of the circular economy and support EU climate energy policy goals.

## 2. Biodegradable Materials in Additive Manufacturing Technologies

One popular solution is to use biodegradable materials in additive manufacturing technologies. Biodegradability is defined as a material’s ability to decompose through the action of microorganisms. The degradation and disposal of non-biodegradable materials, such as plastics, can take hundreds of years. Improper management of these materials leads to pollution and environmental degradation [37]. Currently, 3D printing relies primarily on non-biodegradable materials that are difficult to dispose of, threatening waste management systems. To overcome these limitations, research on various types of bio-based materials has been initiated [7,38]. Biodegradable materials are most often derived from renewable sources, such as corn starch or sugar. These materials emit fewer volatile organic compounds (VOCs) during the printing process, thereby reducing environmental impact. These materials naturally degrade and do not contribute to landfill waste or environmental contamination [37]. Currently, only a few materials are fully biodegradable and suitable for 3D printing. Examples include polylactic acid (PLA) and polyhydroxyalkanoates (PHAs). Their properties can be modified by adding substances, such as fibers or filler [37]. Blends containing polysaccharides (e.g., starch) lower production costs and accelerate degradation. Adding cellulose fibers improves stiffness and resistance to high temperatures. However, due to their limited availability, biodegradable materials are typically more expensive than conventional materials [7,37,38]. Additionally, they generally have lower tensile strength and are more brittle than traditional plastics. Despite their limitations, natural, biodegradable polymers are growing in popularity in additive manufacturing. They demonstrate strong potential as additives for modifying polymer properties. One effective method for improving the physical properties of commonly used materials, such as PLA or ABS, is to reinforce polymers with bio-fibers or combine them with other biodegradable components. Polymers can also be enhanced with fillers to improve their thermomechanical properties. Using natural fillers achieves biodegradability, biocompatibility, and reduced environmental impact [7]. Furthermore, this process influences the physical and mechanical characteristics of the final product, making raw materials more accessible and reducing production costs (Table 5).

### Application of Thermoplastic Starch (TPS) in Thermal Insulation Materials Produced by Additive Manufacturing (3D Printing)

Thermoplastic starch (TPS) is a popular 3D printing material, especially in polymer blends. Obtained from commonly grown industrial crops such as cassava, corn, and potatoes, TPS offers a more environmentally friendly alternative to petroleum-based polymers [94]. It is produced by plasticizing natural starch, a renewable polysaccharide extracted from agricultural feedstocks. TPS is a biodegradable thermoplastic. Using starch derived from agricultural waste or non-food-grade raw materials improves resource efficiency and supports rural economic development. Traditionally, TPS has been used to produce packaging films and agrotextiles due to its biodegradability and low environmental impact [95]. However, recent advances in research have expanded its potential applications to include 3D printing [79,80]. Currently, low-cost TPS is incorporated into PLA to produce cost-effective PLA/TPS blends. Many studies have examined blending PLA and TPS, and the most important findings are outlined below.

Haryńska et al. [79] were among the first to demonstrate the suitability of polylactide (PLA)/potato starch (TPS) filaments for fused filament fabrication (FFF) and fused deposition modeling (FDM). The researchers conducted a comprehensive study on a new FFF-type 3D printing filament consisting of 60% PLA and 40% TPS, with epoxidized soybean oil serving as a modifier. The researchers produced the PLA/TPS filament via extrusion and compared it with commercial PLA (FlashForge). The researchers demonstrated the structural stability of both materials during the FFF process. The PLA/TPS filament exhibited a higher degree of crystallinity prior to printing (46.3%), which decreased to 17.4% during 3D printing. Degradation proceeded in two stages (TPS and PLA). Its thermal stability was slightly lower than that of PLA. PLA/TPS showed higher melt flow at elevated temperatures, which limited the printing temperature to approximately 190–200 °C. The authors determined the dependence on build orientation and raster angle. The ZX_0° configuration produced the best results. PLA/TPS exhibited a tensile strength of ~18 MPa and a Charpy impact strength of ~9.7 kJ/m^2^. Although PLA/TPS is more brittle than PLA, it exhibits better ductility when correctly oriented. Compressive strength increased with infill density, reaching ~30 MPa at 100% infill. The PLA/TPS produced by the authors had a more hydrophilic surface (contact angle of 63–81°), was more susceptible to hydrolytic degradation (experiencing greater mass loss in PBS than pure PLA), and lost 19% of its mass under composting conditions. In contrast, the commercial PLA remained intact. The authors successfully 3D-printed anatomical models (L3 and C1 vertebrae) and complex porous structures (gyroid and sponge-like), confirming the material’s practical suitability.

The next researchers to develop cost-effective TPS/PLA/PBAT filaments for FDM were Ju et al. [80]. They used a two-step approach to expand the material palette to include more flexible TPS-based systems. The researchers prepared blends consisting of 50% TPS, 40% PLA, and 10% PBAT. They enhanced the blends’ mechanical properties by incorporating a CE. The TPS used was obtained from corn starch that was plasticized with glycerol. TPS/PLA/PBAT blends with various CE contents (0–1%) were produced by extrusion. Some of the material was processed into plates via hot pressing, and the rest was processed into 1.75 mm filaments for 3D printing. The researchers demonstrated that adding a CE increased the melting and crystallization temperatures of PLA and stabilized its crystallinity. As the CE content increased, the melt viscosity and rheological modulus rose, thereby improving printing stability. Scanning electron microscopy (SEM) analyses revealed that the CE enhanced adhesion between the PLA and PBAT at the interface. This reduced the domain size and improved compatibility. Furthermore, increasing the CE content decreased the melt flow index, resulting in better print control. The fabricated TPS/PLA/PBAT filaments produced accurate prints. CE decreased porosity and improved surface smoothness. It also increased the elongation at break by 113% and the impact strength by 190% for hot-pressed specimens. Similar improvements were observed for FDM-printed parts; however, their values were lower than those of hot-pressed samples due to weaker interlayer adhesion.

Qin et al. [81] proposed a one-step preparation method for PLA/TPS blends using pyrogallol acid (PGA) as a compatibilizer at concentrations ranging from 0 to 2 parts per 100 (phr). However, since the study focused on blends rather than printable filaments, its application to fused deposition modeling (FDM) was not examined. The researchers demonstrated that adding 1.5 phr of PGA produced optimal results, including a tensile strength of 23.38 megapascals (MPa) and an elongation at break of 16.96%. These values increased by 24.7% and 233.2%, respectively, compared with the pure PLA/TPS blend. Scanning electron microscopy (SEM) revealed improved interfacial adhesion and more homogeneous morphology. Differential scanning calorimetry (DSC) and thermogravimetric analysis (TGA) confirmed that the PGA-containing blends exhibited increased crystallinity and thermal stability. DMTA indicated a higher storage modulus and activation energy, demonstrating better compatibility. Contact angle and moisture sorption tests showed that water resistance increased significantly with the addition of PGA.

Li & Huneault [77] conducted a classic study comparing the use of glycerol and sorbitol as plasticizers for TPS in TPS/PLA systems. They varied the plasticizer content from 30 to 42% and the TPS content from 27 to 60%. In all blends, the PLA formed the continuous phase, while the TPS formed the dispersed phase. The blends were produced by twin-screw extrusion and were analyzed for morphology, mechanical properties, and thermal properties. The study revealed that the glycerol-to-sorbitol ratio significantly impacted the properties. Sorbitol-plasticized blends exhibited finer morphology, higher tensile strength and elastic modulus, and lower crystallization rates. These results confirm that the properties of TPS/PLA composites can be tailored by selecting an appropriate plasticizer.

Müller et al. [96] described the interactions and morphology of poly(lactic acid)/plasticized starch (PLA/PS) blends. TPS was prepared from corn flour by adding either 36% or 47% glycerol. PLA/TPS blends were formulated at various volume fractions (0–100%). The authors demonstrated that PLA and TPS were immiscible and formed a two-phase structure at every stage. They also showed that glycerol primarily remained in the TPS phase, diffusing minimally into the PLA phase. Modeling indicated slight PLA dissolution in TPS (up to ~3 vol.%), whereas TPS did not dissolve in PLA. Scanning electron microscopy (SEM) revealed heterogeneous structures. PLA was the continuous phase at low TPS content, and TPS was the continuous phase at high TPS content. Only a co-continuous structure was obtained within a narrow range around 50%. As the TPS fraction increased, the elastic modulus and strength decreased. TPS markedly degraded the properties of PLA, resulting in low strength and limited ductility. DMA analysis and modeling revealed weak interfacial adhesion and ineffective stress transfer. Additionally, the authors demonstrated that dispersed glycerol droplets formed at glycerol concentrations greater than 1–2 vol.%. This finding was later used to interpret the microstructures presented in this article.

Xiong et al. [97] demonstrated the presence of epoxidized soybean oil (ESO) microdroplets in polylactic acid (PLA), which was due to the plasticizing effect of ESO. This analogy was later used to discuss the morphology of PLA/tributyl citrate (TBC)/glycerol systems. The authors prepared modified starch (MGST) by grafting maleic anhydride (MA) groups, then produced PLA/starch, PLA/ESO, PLA/ESO/starch, and PLA/MGST/ESO blends via co-extrusion. PLA alone was brittle, with an elongation at a break of ~5%. Adding ESO lowered the Tg and Tc of PLA, acting as a plasticizer. However, combining PLA with unmodified starch did not yield compatibility. Pronounced phase boundaries were observed, and the mechanical properties deteriorated. For the PLA + starch + ESO blend, however, interfacial adhesion improved and elongation at break increased to 64%. Impact strength also increased to ~30 kJ/m^2^. The blend with the best performance was the 80/10/10 PLA + MGST + ESO blend. It had an elongation at a break of 140% (28 times greater than pure PLA) and an impact strength of 42 kJ/m^2^ (more than twice that of pure PLA). It also had an acceptable tensile strength of ~43 MPa. Scanning electron microscopy (SEM) revealed that a higher degree of MGST modification resulted in the disappearance of phase boundaries and improved starch dispersion within the PLA matrix. The optimal effect was observed at a moderate ESO content of ~10 wt%; however, excess ESO (>12–15%) caused a decline in mechanical performance due to over-plasticization.).

Chang, Trinh, & Mekonnen [95] studied multilayer TPS/PLA films that exhibited excellent gas and moisture barrier properties. Although these films are not filaments, the study clearly illustrates how material architecture can improve the performance of PLA/TPS systems. The authors prepared monolayer films (TPS, MTPS, and PBAT, as well as their blends) and multilayer films with PBAT coatings (TPS/MTPS cores with PBAT outer layers). The process involved reactive extrusion, compression molding, and dip coating. Modifying TPS (MTPS) improved its compatibility and adhesion with PBAT due to the presence of ester groups and transesterification reactions. MTPS/PBAT films exhibited a more homogeneous phase distribution than unmodified TPS/PBAT films. Incorporating PBAT improved the films’ thermal resistance, increasing their strength and modulus. The multilayer film (TPS/MTPS with PBAT coatings) demonstrated even better performance. Interlayer adhesion strengthened due to MTPS compatibilization and PBAT–PBAT interactions. This resulted in a transition from interfacial (delamination) failure to cohesive failure. Additionally, water vapor transmission resistance increased by up to 86.8%, and oxygen permeability decreased by 65.6–74.3% compared with pure PBAT.

Jiang et al. [94] conducted a study to develop a new one-step method of producing PLA/TPS composite filaments for 3D printing. The researchers achieved a homogeneous dispersion of the starch granules within the PLA matrix. However, they also observed pores, indicating weak interfacial bonding. At a high glycerol content, microdroplets of the plasticizer appeared in the PLA. The researchers confirmed the complete plasticization of the starch with no significant changes in PLA crystallinity. DSC and TGA analyses revealed a decrease in the cold crystallization temperature from 128 °C for pure PLA to 104–110 °C for the composites. Introducing TPS did not diminish the thermal stability of the PLA. However, additional TPS and glycerol degradation was observed. The melt flow index (MFI) increased with the rising TPS content, which improved print quality by reducing interlayer voids. Additionally, the contact angle decreased by ~20° at 10% TPS content, indicating increased hydrophilicity due to the presence of hydroxyl groups. The PLA/TPS composites produced by the authors exhibited a slightly greater mass loss than pure PLA. However, degradation remained very slow (≤0.1%). Tensile strength decreased from 49.5 MPa for pure PLA to 38.6 MPa for 10% TPS. Nevertheless, these values exceeded those of some common plastics (e.g., HDPE). Elongation at break increased slightly with a higher TPS/glycerol content. Honeycomb-structured specimens made from PLA/TPS filaments had porosity and compressive strength comparable to trabecular (spongy) bone, indicating their potential for biomedical applications. The filaments were used to 3D print mechanical test specimens, porous structures, and demonstration models, all of which had smooth surfaces with no visible defects. Adding TPS improved dyeability with ordinary and fluorescent inks, enabling decorative and anti-counterfeiting features.

Currently, applications of PLA/TPS blends rely solely on the FDM additive method. However, the aforementioned studies (Table 5) indicate that natural biodegradable substances can reinforce composites manufactured by selective laser sintering (SLS). These substances include powders obtained from rice husks, pine wood, phenolic resin, keratin, and wool combined with popular polymers such as nylon (PA11 and PA12). Studies have demonstrated that using natural fibers in SLS increases biodegradation and significantly improves thermal stability and mechanical properties, including tensile and flexural strength.

Therefore, this study aims to develop modern thermal insulation materials from PA12/TPS blends with thermal conductivity comparable to that of conventional materials. These materials could improve the energy efficiency of buildings.

Accordingly, this project involves using 3D printing technology and the selective laser sintering (SLS) method to develop prototypes of thermal insulation materials based on Kelvin cell structures. These materials will be produced in one step from a composite of biodegradable polyamide (PA12) and thermoplastic starch (TPS). A second objective is to determine the relationships between the thickness and layering of the composites, the printing material used, and the resulting thermal properties, such as thermal conductivity, thermal resistance, and thermal transmittance, through experimentation. Finally, we will evaluate the effect of the fabricated partition on the energy performance of an energy-efficient building. The building will undergo an energy balance analysis using ArCADia TERMO 11 software for energy audits. Energy demand calculations will be performed for partition variants with the most favorable insulation properties

## 3. Materials and Methods

**Figure 1:**
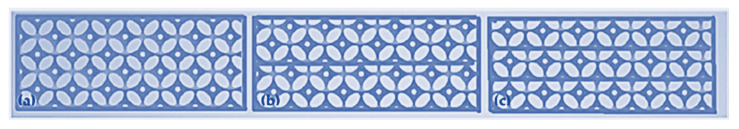
Layer arrangement for a 20 mm thick composite: (**a**) single layer, (**b**) double layer, and (**c**) triple layer [68].

**Figure 2:**
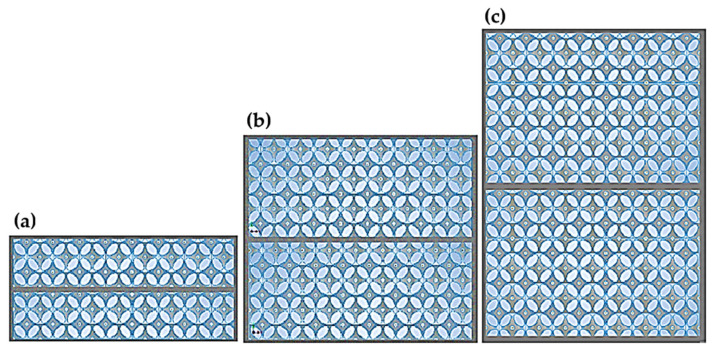
Layer arrangement for a double-layer composite: (**a**) 20 mm (**b**) 40 mm, and (**c**) 60 mm.

**Figure 3:**
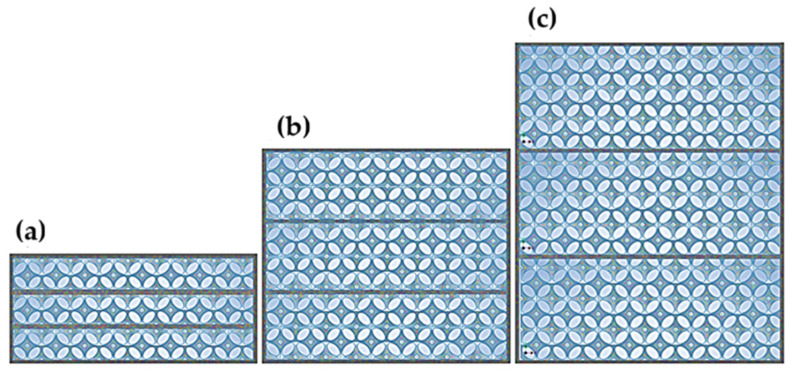
Layer arrangement for a triple-layer composite: (**a**) 20 mm (**b**) 40 mm, and (**c**) 60 mm.

The prototype thermal insulation materials were designed for research purposes. The geometric model of the open-cell insulation is based on the Kelvin cell structure described by the author in a previous publication [68]. The final designs were 3D-printed using selective laser sintering (SLS) with polyamide (PA12) and the empty spaces were filled with air. The resulting composites had different total thicknesses and layering. Three thickness variants (d) were created: 20 mm, 40 mm, and 60 mm. For each of these, three additional configurations consisting of one, two, or three layers were designed. The layer distribution is illustrated in Figure 1, Figure 2 and Figure 3.

The individual layers were separated by spacers with the minimum thickness printable by the Sinterit Lisa 3D printer (Sinterit Company, Krakow, Poland), which is 0.2 mm. A total of 9 models with different geometries were created, as shown in the table (Table 6), where A represents the edge length of the square base, (d) the height, and (n) the number of layers.

**Figure 4:**
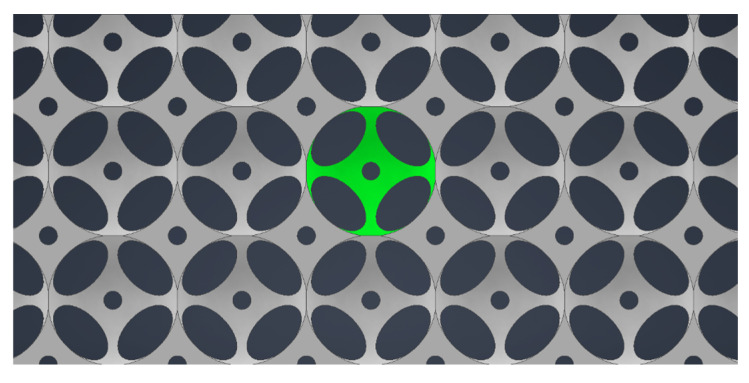
Cyclical repetition of the unit cell structure (own elaboration).

The process of creating the prototype thermal insulation material involved repeatedly forming individual open-cell structures until a solid object with a square base measuring A × A and a height of (d) was formed (Table 1). Figure 4 shows a top view of the open-cell structure, highlighting the unit cell that was repeated to create the prototype thermal insulation material.

**Figure 5:**
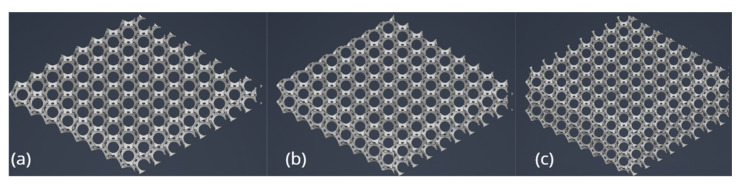
Isometric comparison of layers: (**a**) material forming three layers, (**b**) material forming two layers, and (**c**) material forming one layer (own elaboration).

Figure 5a–c show an isometric view of a comparison of the materials forming the individual layers of the 20 mm variant.

### 3.1. Material for Printing

The prototype thermal insulation materials were produced using a powder mixture of polyamide 12 (PA12) (Sinterit Company, Krakow, Poland) and thermoplastic starch (TPS) (Wroclaw University of Environmental and Life Sciences, Wroclaw, Poland). To examine the influence of the two materials on each other’s thermal insulation properties in 3D prints, three variants of PA12 + TPS mixtures were prepared with different weight percentages of powder. These variants are presented in Table 7.

Polyamide 12 (PA12) is one of the most commonly used materials in selective laser sintering (SLS), a type of additive manufacturing. As a semi-crystalline thermoplastic polymer with excellent sintering properties, PA12 is used for 3D printing various technical applications, including in dentistry and in producing composite materials [98]. The powdered PA12 used in this study was supplied by the manufacturer of the Sinterit Lisa printer used for the experiments. According to the manufacturer, the material is characterized by high precision, biocompatibility, chemical resistance, and good mechanical properties [98]. Furthermore, it can be mechanically recycled by mixing waste powder with fresh powder in subsequent production cycles.

Thermoplastic starch (TPS) is a fully biodegradable polysaccharide primarily used as an additive in biodegradable polymer composites. Using TPS can reduce production costs and modify the physical properties of materials, including their biodegradability, heat resistance, and 3D printability. However, due to its limited mechanical properties, such as brittleness and accelerated ageing, pure TPS is not suitable for direct use in 3D printing [10]. To enhance its performance, plasticisers, reactive modifiers, and chemical starch modification are often used, as is blending with other polymers.

**Figure 6:**
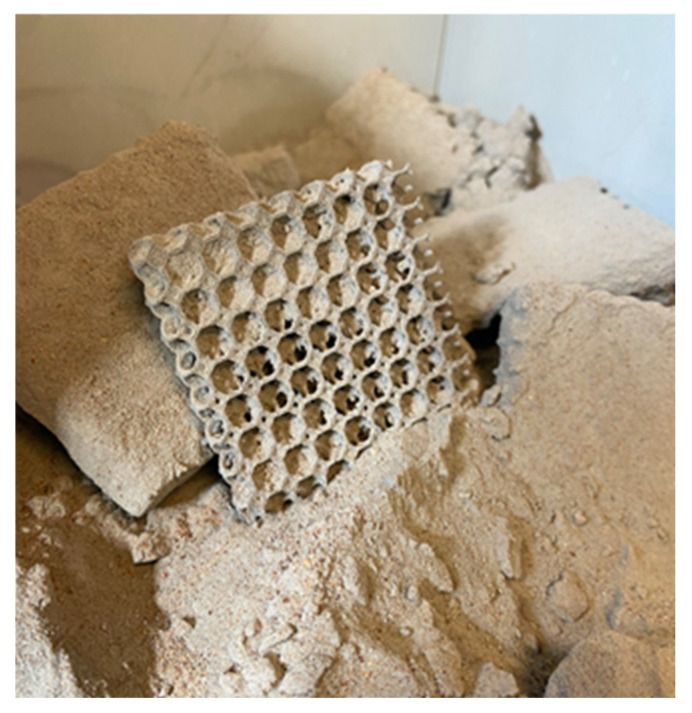
Preliminary cleaning (own design).

**Figure 7:**
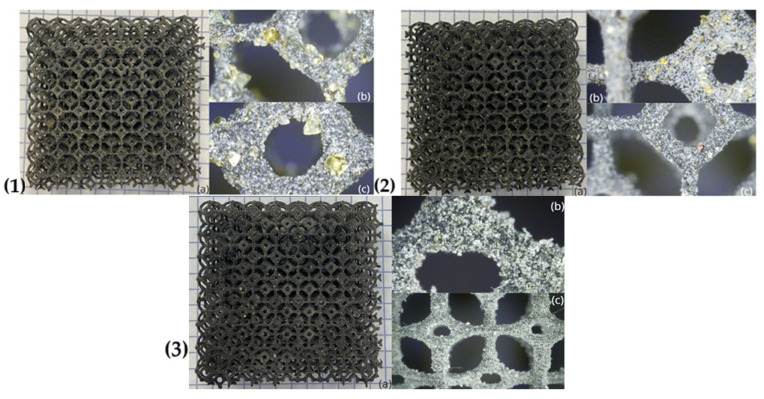
(**1**) The structure of the prototype thermal insulation material with a PA12:TPS mass ratio of 3:7 (**1a**); (**1b**,**1c**) a microscopic images of the PA12:TPS (3:7) structure. (**2**) The structure of the prototype thermal insulation material with a PA12:TPS mass ratio of 5:5 (**2a**); (**2b**,**2c**) a microscopic image of the PA12:TPS (5:5) structure. (**3**) The structure of the prototype thermal insulation material with a PA12:TPS mass ratio of 7:3 (**3a**); (**3b**,**3c**) a microscopic image of the PA12:TPS (7:3) structure (own elaboration).

**Figure 8:**
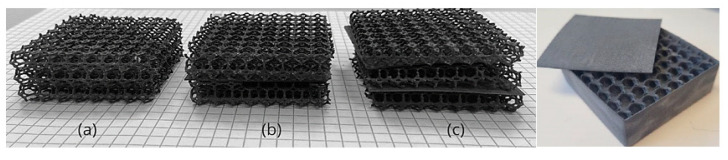
Prototype thermal insulation material with a thickness of 20 mm: (**a**) single layer, (**b**) double layer, (**c**) and triple layer (own elaboration); thermal insulation print casing (to the right, as shown in the picture).

As part of the work on designing and printing the prototype thermal insulation materials, a total of 27 structures were created. These structures were made using different proportions of the raw material (PA12 + TPS) and varied in size (d = 20/40/60 mm) and the number of layers (n = 1–3). All of the fabricated structures are presented in Table 7. Figure 6, Figure 7 and Figure 8 show photographs and microscopic images of the manufactured prototype insulation material structures, while Figure 8 illustrates layering for a 20 mm thick material.

### 3.2. Experimental Determination of the Thermal Transmittance Coefficient

**Figure 9:**
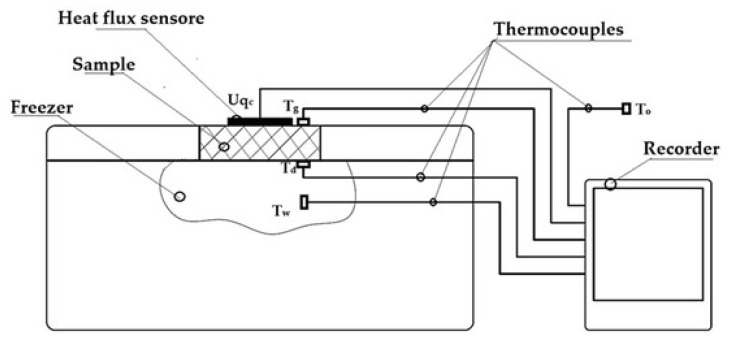
Schematic of the test stand for thermal insulation testing [65,68,70].

All variants of the prototype thermal insulation materials described above, which were manufactured using selective laser sintering (SLS) 3D printing technology, were subjected to experimental testing to determine their thermal conductivity coefficient (λ), thermal resistance (R), and thermal transmittance coefficient (U). These measurements were performed in accordance with ISO 9869-1:2014 [99] using an existing test stand at the Department of Energy Conversion Engineering at Wrocław University of Science and Technology’s Faculty of Mechanical and Power Engineering [65,68,70]. A schematic diagram of the test rig is shown in Figure 9.

During the measurements, the samples were placed in a hole in the lid of an Aisberg LP15 C15 freezer (MELIS, Poznań, Poland) so that the bottom of each sample was in direct contact with the inside of the freezer and the top with the outside. A frame measuring 340 × 265 × 20 mm was constructed in place of the lid to accommodate samples measuring 60 × 60 × 20 mm. During a test, four samples of different types were placed simultaneously in the area of the freezer lid.

The mechanism of heat flow through the specimen was based on the temperature difference between the environment (outside) and the inside of the freezer. The heat flux density through the insulation under test was measured using an FHF04SC sensor (Hukseflux Thermal Sensors B.V., Delft, The Netherlands) and the data were recorded on a recorder every 0.5 min. During the measurements, temperatures were measured at the following locations: on the outside surface of the sample, on the inside surface of the sample, inside the fridge/freezer, and around the outside of the fridge/freezer (see location of thermocouples in Figure 8). Temperatures outside the sample were assumed to be +20 °C (on the ambient side) and −20 °C (in the refrigerator/freezer compartment) due to the typical operating conditions of thermal insulation of buildings, the food industry, and the transport of frozen foods. The accuracy of the measuring instruments is given in Table 8.

For these boundary conditions, the thermal insulation of the materials was measured at an average sample temperature of 0 °C. The measured values were used to calculate the thermal conductivity coefficient λ and the thermal resistance R. The measured values were recorded after thermal equilibrium had been reached. This state was considered to have been reached when the temperature variation at the surface of the test specimens did not exceed 0.5 °C for successive readings over a period of 1 h.

### 3.3. Quantitative Method for Calculating Thermal Parameters

The methodology for quantifying the thermal parameters was based on measuring the electrical voltage and converting it into heat density flux according to Equation (1) specified by the device manufacturer [22,65,70].

(1) $$q=\frac{U_{qc}}{0.0103}$$

where

- *q* is the heat flux density, [W/m^2^];
- *Uqc* is the voltage of the flowing current, [mV].

At the same time, the temperatures on the top (hot) and bottom (cold) surfaces of the test samples, as well as the air temperature inside and outside the cold chamber, were measured on the test bench. These temperatures were measured using K-type thermocouples. Based on the measured temperatures and the heat flux density during the steady-state phase of heat flow through the sample, the heat transfer coefficient was calculated using Equation (2) [69,70].

(2) $$\lambda =\frac{d\cdot q}{T_{g}-T_{d}}$$

where

*λ* is the design thermal conductivity of the material, [W/m·K];
*d* is the thickness of the test sample, [m];
*q* is the heat flux density, [W/m^2^];
*T_g_* is the temperature of the upper surface of the sample, [°C];
*T_d_* is the temperature of the lower surface of the sample, [°C].

Subsequently, the heat transfer coefficient U was estimated for the material thicknesses determined according to the methodology specified in ISO 6946 [100], as well as for the homogeneous material partitions. The calculations assumed a horizontal direction of heat transfer, as for vertical external partitions (walls). This assumption allowed for the selection of appropriate thermal resistance coefficients for the internal air layers *R_si_* = 0.13 and the external *R_se_* = 0.04. The U-value was determined according to Equation (3) [70].

(3) $$U=\frac{1}{R_{si}+\frac{d_{i}}{\lambda_{i}}+R_{se}}$$

where

*U* is the thermal transmittance, [W/m^2^·K];
*R_si_* is the internal surface resistance, [m^2^·K/W];
*R_se_* is the external surface resistance, [m^2^·K/W];
*d_i_* is the thickness of the material layer (i) in the component, [m];
*λ_i_* is the design thermal conductivity of the material layer (i), [W/m·K].

## 4. Results and Discussion

Statistical analyses were conducted using STATISTICA 13 software (TIBCO Software Inc., Palo Alto, CA, USA). In line with standard procedures in thermal insulation research, a significance level of *p* ≤ 0.05 was adopted. Measures of central tendency and dispersion were determined, and their summary results are presented in Table 3. Analysis of the *p*-values in Table 9 indicates that values below 0.05 demonstrated a statistically significant impact of the experimental input variables on the thermal properties of materials produced using selective laser sintering (SLS) 3D printing technology.

In summary, to optimize the thermal insulation properties of the manufactured composites, it was possible to determine the most favorable proportion of the natural TPS additive (% PA12:TPS), the optimal composite thickness (d), and the optimal number of layers (n). Each input variable was independently adjusted.

The analysis of variance (Table 8) revealed that the amount of TPS added to polyamide PA12, variation in composite thickness (d), and number of layers (n) affected the thermal properties of the insulation barriers produced using 3D printing technology, as confirmed by the *p*-value. The statistical significance of the linear factors was also confirmed by the large effect size (F-value). Compared with other input variables, the dominant factors were the thickness and layering of the produced composite, as well as the amount of TPS added to the composite structure. This study also confirmed the statistical significance of interactions between linear factors. It was found that composite thickness clearly dominated over the other input variables.

A ranking was performed to identify the dominant factors based on their strength of influence (F) on the model and their interactions. As shown in Table 8, specimen thickness (both total thickness and layer thickness for specific specimen types) was a dominant factor (F = 3700.44, *p* < 0.001, η^2^ = 0.993), surpassing the other input variables. A significant main effect was also found for the number of layers (n) (F = 232.75, *p* < 0.001, partial η^2^ = 0.896). Similarly, composition (%_PA12) had a significant effect (F = 90.39, *p* < 0.001, partial η^2^ = 0.770); however, its effect was much smaller than that of the aforementioned input factors. The pattern of simple effects indicates that increasing d systematically reduces λ.

Additionally, the d × n interaction was found to substantially impact the thermal conductivity coefficient: Type II: F = 85.88, *p* < 0.001, partial η^2^ = 0.864. This result remained stable under Type III sums of squares as well: F = 25.95, *p* < 0.001, partial η^2^ = 0.658. The d × n interaction remained significant at every composition level (%_PA12): 30.0: F = 39.46, *p* < 0.001, partial η^2^ = 0.898; 50.0: F = 14.67, *p* < 0.001, partial η^2^ = 0.765; and 70.0: F = 72.24, *p* < 0.001, partial η^2^ = 0.941. Increasing n significantly reduced λ for d = 20–40 mm. However, at d = 60 mm, the number of layers no longer affected conductivity because there were no significant differences.

Example mean λ values (averaged over composition) were as follows: (i) n = 1: d = 20 → 0.0482, d = 40 → 0.0433, and d = 60 → 0.0291 (a drop of 0.0190 from 20 to 60); (ii) n = 2: d = 20 → 0.0443 and d = 40 → 0.0397, (iii) n = 3: d = 20 → 0.0401, d = 40 → 0.0397, d = 60 → 0.0291 (a drop of 0.0110).0396, and d = 60 → 0.0287 (a drop of 0.0156); and (iii) n = 3: d = 20 → 0.0401, d = 40 → 0.0397, and d = 60 → 0.0291 (a drop of 0.0110).

For d = 20 mm and d = 40 mm, Tukey tests confirmed significant differences between n levels; for d = 60 mm, no differences between n were observed. In summary, it can be concluded that sample thickness (d) is the strongest lever for reducing λ, and increasing the number of layers (n) is worthwhile mainly for thinner specimens—namely d = 20 mm and d = 40 mm—whereas, at d = 60 mm, additional layers do not provide further thermal benefits. The effects are robust with respect to composition (%_PA12), but the %_PA12 × d and %_PA12 × n interactions indicate that the parameter settings should be tailored to the specific composition.

### 4.1. Analysis of the Test Results for the Thermal Properties of Thermal Insulation Materials

**Figure 10:**
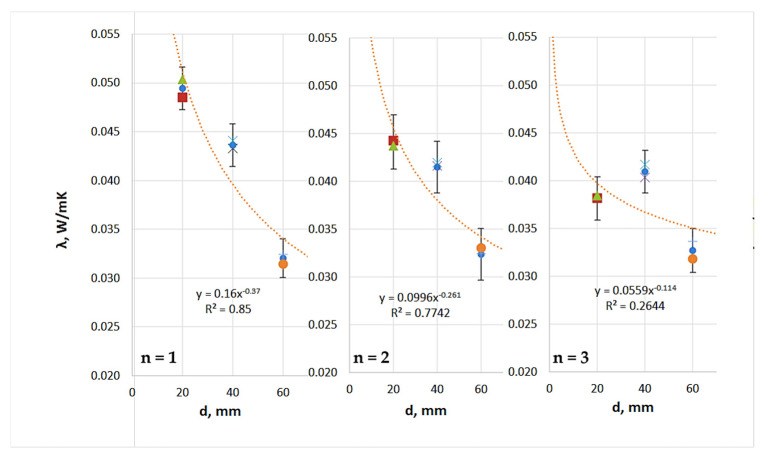
Influence of thickness (d) and number of layers (n) on the thermal conductivity coefficient (λ) of prototype thermal insulation materials with a TPS:PA12 mass ratio of 30:70 (own work).

Analysis of the thermal insulation parameter measurements for the TPS + PA12 prototype composites (Table 10 and Figure 10) revealed the following: (i) the average thermal conductivity coefficient (λ) ranged from 0.049 to 0.032 W/(m·K); (ii) the thermal resistance (R) ranged from 0.406 to 1.867 (m^2^·K)/W; and (iii) the thermal transmittance coefficient (U) ranged from 0.536 to 2.461 (W/(m^2^·K)). Of the TPS + PA12 materials with a 30:70 mass ratio, the single-layer composite with a thickness of 60 mm exhibited the best thermal insulation performance, with the lowest thermal conductivity and thermal transmittance, and thus the highest thermal resistance. The least effective material was the single-layer composite with a thickness of 20 mm.

**Figure 11:**
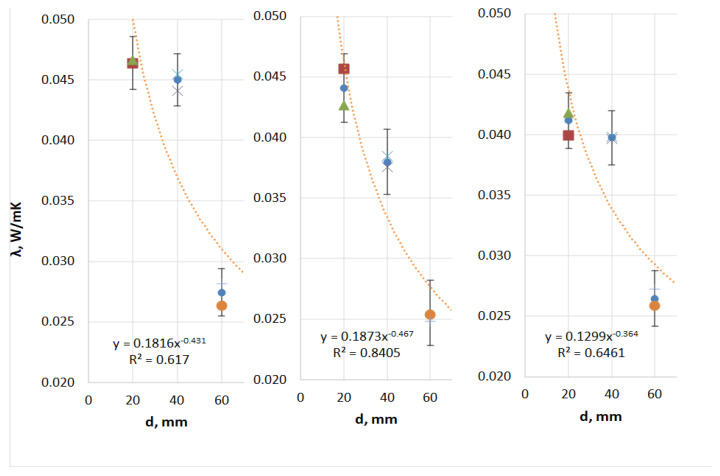
Influence of thickness (d) and number of layers (n) on the thermal conductivity coefficient (λ) of prototype thermal insulation materials with a TPS + PA12 mass ratio of 50:50 (own work).

Analysis of the thermal insulation parameter measurements for the TPS + PA12 prototype composites (Table 11 and Figure 11) revealed the following: (i) the average thermal conductivity coefficient (λ) ranged from 0.046 to 0.026 W/(m·K); (ii) the thermal resistance (R) ranged from 0.433 to 2.360 (m^2^·K)/W; and (iii) the thermal transmittance coefficient (U) ranged from 0.424 to 2.311 (W/(m^2^·K)).

Of the TPS + PA12 materials with a 50:50 mass ratio, the double-layer composite with a thickness of 60 mm exhibited the best thermal insulation performance. This material showed the lowest thermal conductivity (λ) and thermal transmittance (U), and thus the highest thermal resistance (R). The least effective material was the single-layer composite with a thickness of 20 mm.

**Figure 12:**
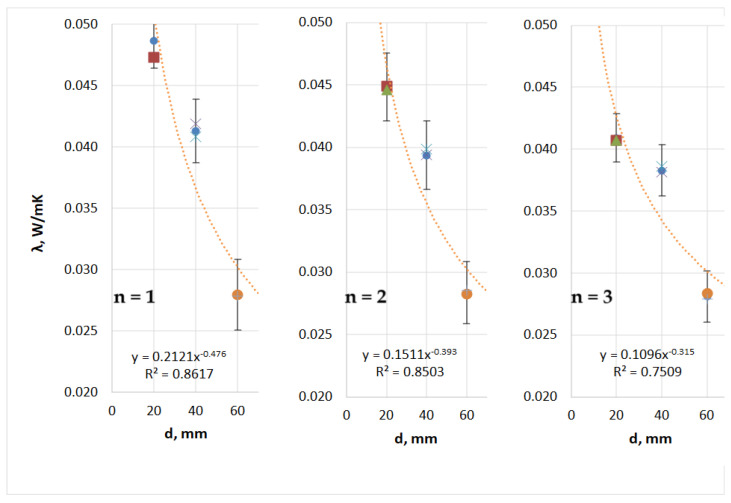
Influence of thickness (d) and number of layers (n) on the thermal conductivity coefficient (λ) of prototype thermal insulation materials with a 70:30 TPS:PA12 mass ratio (own work).

Analysis of the thermal insulation parameter measurements for the TPS + PA12 prototype composites (Table 12 and Figure 12) revealed the following: (i) the average thermal conductivity coefficient (λ) ranged from 0.049 to 0.028 W/(m·K); (ii) the thermal resistance (R) ranged from 0.413 to 2.152 (m^2^·K)/W; and (iii) the thermal transmittance coefficient (U) ranged from 0.465 to 2.420 W/(m^2^·K).

Of the TPS + PA12 materials made in a 7:3 mass ratio, the 60 mm single-layer composite exhibited the best thermal insulation performance. This composite had the lowest thermal conductivity (λ) and thermal transmittance (U) and therefore the highest thermal resistance (R). The least effective material was the single-layer composite with a thickness of 20 mm.

Figure 9, Figure 10 and Figure 11 illustrate the impact of thickness (d) and layering (n) on the thermal conductivity coefficient of the thermal insulation material. Regardless of the mass ratio of the raw material or the number of layers, all TPS + PA12 materials demonstrated a reduction in thermal conductivity (λ) as the thickness of the material increased. The lowest λ values were observed for the largest thickness tested (d = 60 mm), while the highest λ values were recorded for the smallest thickness (d = 20 mm).

**Figure 13:**
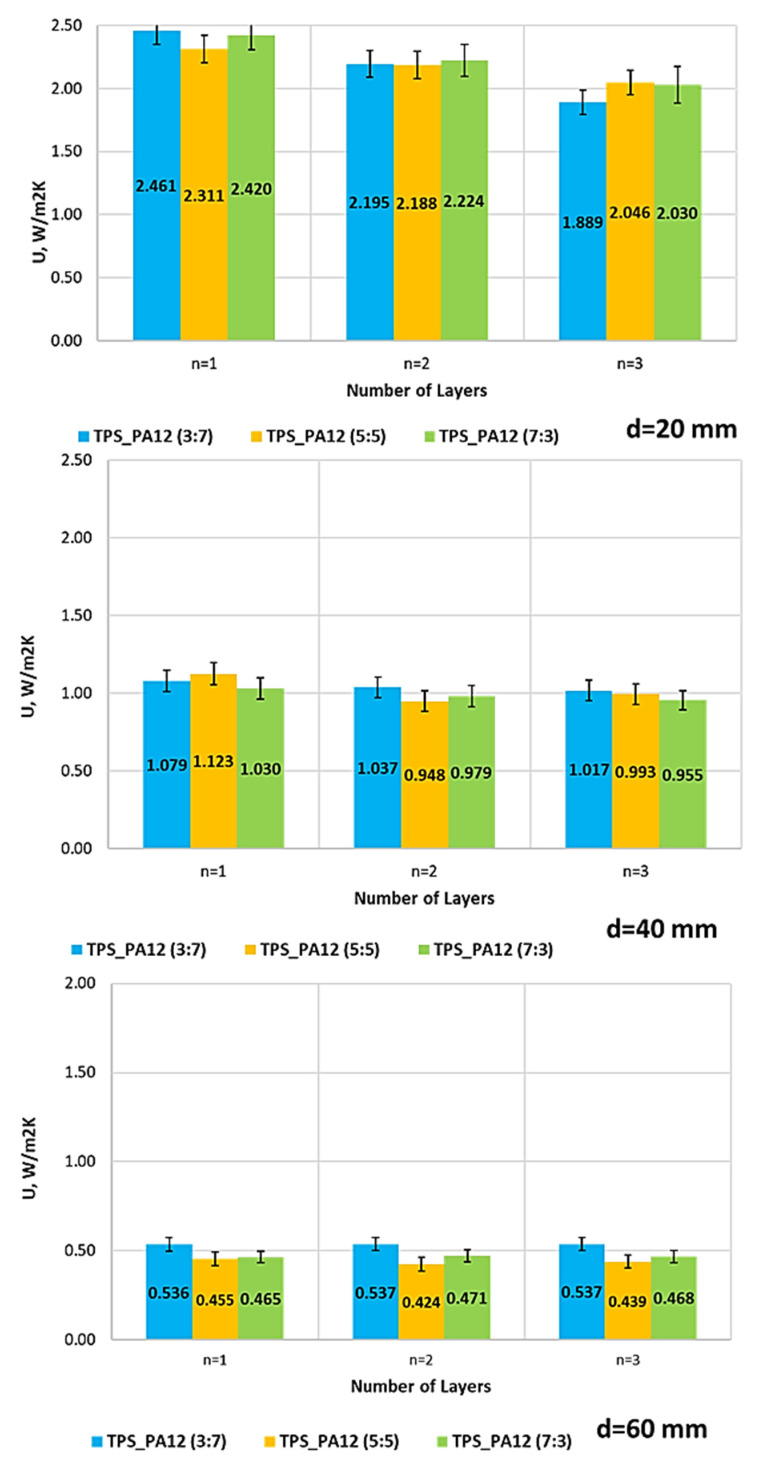
Coefficient of thermal transmittance (U, W/m^2^K) of partitions manufactured from TPS_PA12 composites with different material ratios (3:7, 5:5, 7:3), depending on the number of layers (n = 1–3) and partition thickness (d = 20, 40, 60 mm). Error bars represent standard deviations (original work).

Figure 13 illustrates the impact of thickness, the number of layers, and theTPS + PA12 mass ratio on the thermal transmittance coefficient (U) of prototype thermal insulation materials. An increase in insulation material thickness was found to lead to a decrease in thermal transmittance (U) for all samples, regardless of the material composition or the number of layers.

The results presented in Figure 13 show that the thermal transmittance coefficient (U) was influenced by partition thickness, number of layers, and the TPS:PA12 ratio. For the thinnest partitions (d = 20 mm), the U-values ranged from 1.889 to 2.461 W/(m^2^·K). A systematic reduction in U-value was observed as the number of layers increased. The lowest U-value was obtained for the TPS:PA12 (3:7) sample at n = 3, demonstrating the significant role of the number of layers in improving thermal insulation. At a thickness of d = 40 mm, the U-values decreased by more than half, reaching 0.948–1.123 W/(m·K). The effect of the number of layers became less pronounced; however, the TPS:PA12 (5:5) composition at n = 2 exhibited the lowest transmittance (U = 0.948 W/m^2^K). For the thickest samples (d = 60 mm), U-values decreased further, approaching levels typical of insulating construction materials (0.424–0.537 W/m^2^K). Here, the number of layers had little effect on U; the material composition was slightly more relevant. The lowest value (0.424 W/m^2^K) was recorded for TPS:PA12 (5:5) at n = 2.

Overall, this study confirms that partition thickness dominantly affects thermal insulation. The number of layers primarily affects thin walls (d = 20 mm), while material composition influences thicker elements more. Notably, samples with an equal TPS and PA12 content (5:5) exhibit the best thermal performance at larger thicknesses.

### 4.2. The Application of Prototype Thermal Insulation Materials in Energy-Efficient Construction Is Analyzed

The final stage of the research into the effectiveness of prototype thermal insulation materials involved analyzing the use of composites in energy-efficient construction using ArCADia-TERMO 11 software. This program is used for thermal building calculations and for preparing related documentation, including energy audits and performance certificates. ArCADia-TERMOCAD is the most popular software on the Polish market for creating energy performance certificates, which are required when leasing or selling buildings or premises. It can also be used to calculate the heating and cooling requirements of rooms. The software is also certified for BREEAM calculations.

The analysis aimed to compare the thermal parameters of a single-family residential building insulated using different materials.

The comparison included four variants of external wall insulation as follows:

1. The existing building, where the external walls are insulated with 12 cm thick polystyrene;
2. A building in which the polystyrene has been replaced with a three-layer prototype thermal insulation material with the same thickness (12 cm), made from TPS + PA12 at a 3:7 mass ratio;
3. A building in which the polystyrene has been replaced with a three-layer prototype thermal insulation material consisting of TPS and PA12 at a 5:5 mass ratio and with a thickness of 12 cm;
4. A building in which the polystyrene has been replaced with a three-layer prototype thermal insulation material of the same thickness (12 cm) consisting of TPS and PA12 at a mass ratio of 7:3.

The thermal conductivity values for the 12 cm thick prototype thermal insulation materials were determined based on a power trend chart. Preliminary data analysis indicated that the most suitable model for the relationship was the so-called response surface, i.e., a second-degree (quadratic) polynomial fit, for each of the three cases considered [65]. The calculated thermal conductivity values for TPS + PA12 composites with different mass ratios at a thickness of 12 cm are presented in Table 13. These materials were implemented in the ArCADia-TERMO software.

#### Description of the Analyzed Building

**Figure 14:**
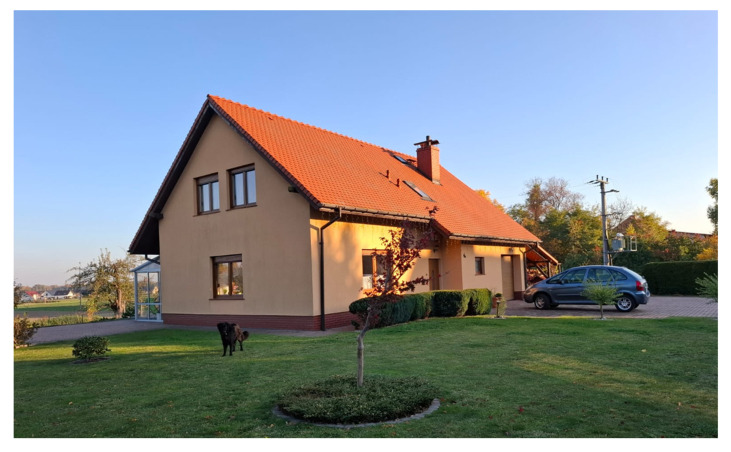

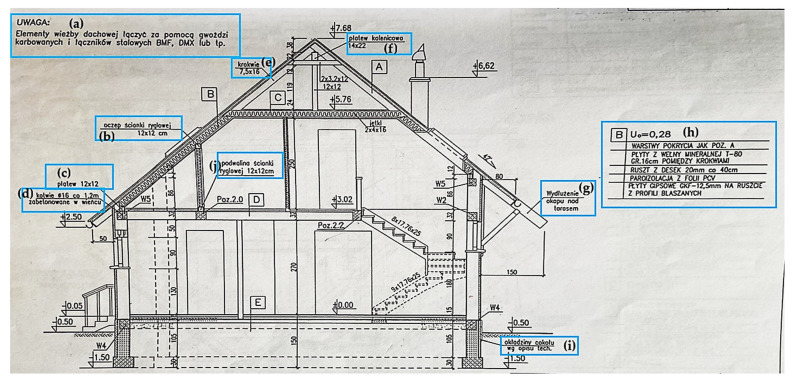
Photograph of the analyzed single-family house. Translation of the designation: (**a**) NOTE: Roof truss elements shall be connected using ribbed nails and steel fasteners such as BMF, DMX, etc., (**b**) cap beam of stud wall 12 × 12 cm, (**c**) purlin 12 × 12, (**d**) anchors Ø16 every 1.2 m embedded in the ring beam, (**e**) rafter 7.5 × 16, (**f**) ridge purlin 14 × 22, (**g**) extended eaves over terrace, (**h**) U_o_ = 0.28, LAYERS AS IN ITEM A, MINERAL WOOL BOARDS T-80, THICKNESS 16 cm BETWEEN RAFTERS GRID FROM BOARDS 20 mm every 40 cm, VAPOUR BARRIER FROM PVC FOIL, GYPSUM BOARDS GKF—12.5 mm ON A METAL PROFILE GRID (**i**) plinth cladding according to technical description, (**j**) sole plate of stud wall 12 × 12 cm. Translation of the designation: A—roof covering layers, B—sloped roof insulated, C—attic floor/ceiling with insulation, D—intermediate floor (above the ground floor), E—floor on the ground.

This study focuses on a single-family residential building that was completed in 2009. It is located in Jerzmanowice, Poland. The two-storey building is used for residential purposes, with living spaces and bathrooms on both the ground and first floors. It also features a partially underground basement, a non-habitable attic, and a garage. The total area of the building is 242.6 m^2^, 156.8 m^2^ of which is usable heated space. Figure 14 shows the building’s design alongside a photograph of the building.

Building and location (main assumptions for simulation): single-family house (occupied in 2009), two stories plus a partial basement, unheated attic, and attached garage. Gross floor area: 242.6 m^2^. Heated floor area: 156.8 m^2^. Location: Jerzmanowice, Poland. Climate station used in the model: Legnica, Poland (climate zone II) with a design outdoor temperature of −18 °C. The heating, ventilation, and air conditioning (HVAC) system uses a gas boiler with radiators, which is typical for the region. The airtightness/air change rate is n_50_ = 3 h^−1^, which is typical for a single-family dwelling with good window and door seals. The baseline (as is) envelope and U-values were modeled using plans and a site inspection. External walls: porotherm 24 cm + EPS 12 cm (actual on site; project assumed 10 cm). The computed U-value is 0.25 W/(m^2^·K). Roof/ceiling to unheated attic (non-homogeneous): U ≈ 0.28–0.29 W/(m^2^·K). Floor above unheated basement: U = 0.30 W/(m^2^·K). Floor on ground: U = 0.29 W/(m^2^·K). Openings (catalog values): windows: U = 1.1 W/(m^2^·K).

External door: U = 1.5 W/(m^2^·K).

Garage door: U = 1.8 W/(m^2^·K). The material properties (λ) used are EPS: 0.040 W/(m·K); mineral wool: 0.038 W/(m·K); and porotherm blocks: 0.283.

Gypsum board: 0.230, etc.

The corresponding modeled wall U-values are 0.21, 0.16, and 0.17 W/(m^2^·K). Surface resistances and thermal bridges: internal and external surface resistances (R_(si) and R_(se)) are set per heat flow direction (e.g., horizontal and vertical flow cases). Representative linear thermal bridges are included with their respective values: roof/wall (R1, 0.55); external corner (C1, −0.05); wall-to-ground (GF1, 0.65); and lintel/sill/jamb (W7, 0.45). All values are in W/(m·K).

All thermal calculations for the building were performed using the ArCADia-TERMO software program. These calculations compared the effectiveness of external wall insulation with that of the baseline variant (Variant 0), in which the walls are insulated with 12 cm thick polystyrene with a thermal conductivity of 0.040 W/m·K. The comparative variants (Variants 1–3) involved replacing the polystyrene with the prototype TPS + PA12 thermal insulation material at different mass ratios.

All of the prototype thermal insulation materials analyzed in ArCADia-TERMO had a lower thermal conductivity coefficient (λ) than the polystyrene used in the real-world scenario. Using the prototype composites instead of polystyrene in each tested material configuration reduced the building’s annual heat demand for space heating and ventilation, as well as decreasing the total design heat load (see Table 14).

In terms of reducing heat loss through external walls, the most effective material was a thermal insulator with a mass ratio of 5:5 of TPS + PA12 (λ = 0.0227 W/m·K). Using this material reduced the building’s total heat load by 0.57 kW, which corresponds to a decrease of 6.37%. Furthermore, it decreased the annual heat demand for space heating and ventilation by 1087.15 kWh/year, resulting in a total reduction of 9.89%.

The TPS + PA12 material with a mass ratio of 3:7 (λ = 0.0324 W/m·K) was the least energy-efficient option. Using this material instead of polystyrene reduced the building’s total heat load by only 0.23 kW (a 2.57% decrease). Additionally, it lowered the annual heat demand for heating and ventilation by just 448.77 kWh/year (a 4.08% reduction).

**Figure 15:**
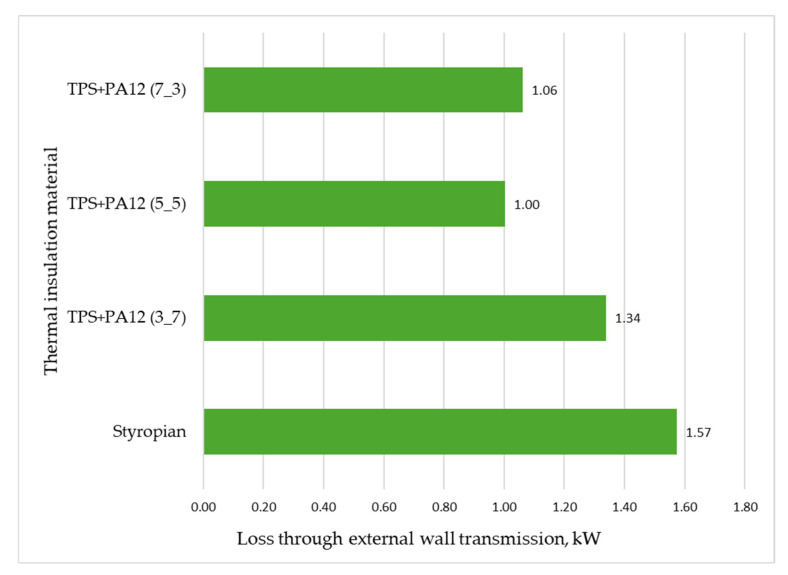
Heat loss through the external walls of the building, depending on the type of thermal insulation material used (own elaboration).

Figure 15 shows how heat loss through the external walls varied when using TPS + PA12 at different mass ratios. Each material was capable of reducing transmission losses. However, TPS + PA12 at a mass ratio of 5:5 provided the best thermal insulation performance, reducing transmission loss through the external walls by 0.57 kW—equivalent to a 36.31% decrease compared with the current insulation.

**Figure 16:**
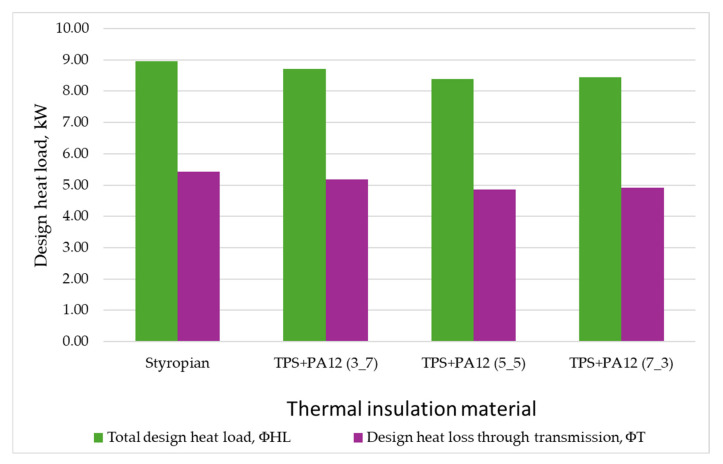
Designed heat load of the building depending on the thermal insulation material applied (own work).

Figure 16 illustrates the proportion of heat lost through transmission as part of the building’s total heat load. For each of the analyzed variants, it was observed that transmission losses accounted for more than half of the total losses contributing to the overall heat load of the single-family house. Reducing these losses decreased the building’s heat load for each insulation variant.

Table 15 shows the heat demand for space heating and ventilation, divided into individual thermal zones. Garage heating had a heat demand of 0 kWh/year in all variants due to minimal heat losses and gains from adjacent rooms.

**Figure 17:**
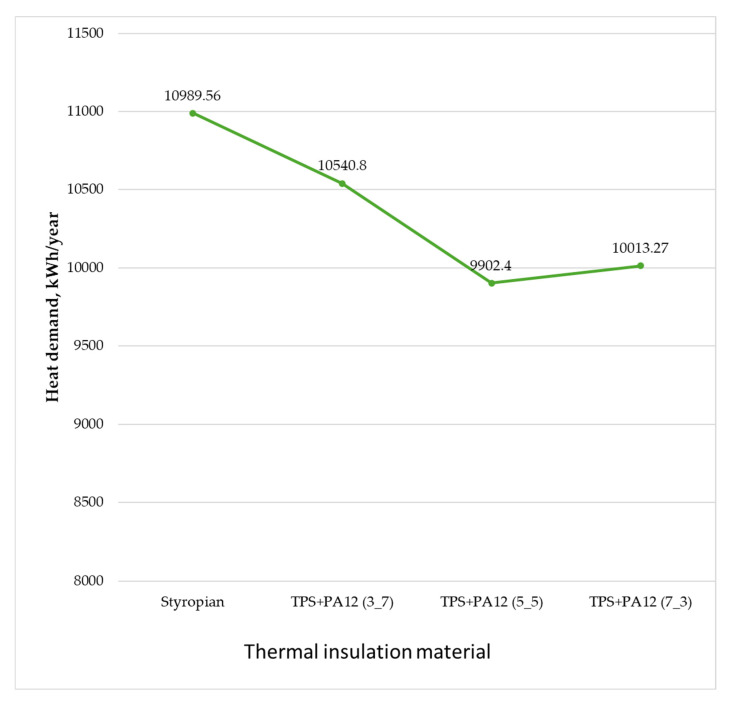
Heat demand of rooms insulated with prototype thermal insulation materials (own elaboration).

Figure 17 illustrates the impact of different insulation materials on the total heat demand for space heating and ventilation. In a real-world scenario, the energy demand amounted to 10.989.56 kWh per year. Each of the manufactured prototype materials reduced the energy demand to a certain extent. The most effective solution was to use TPS + PA12 at a 5:5 ratio, reducing the energy demand to 9.902.41 kWh per year. Using TPS + PA12 at a ratio of 3:7 was the least effective solution, reducing the energy demand to 10,540.80 kWh/year.

Table 16 shows the proportion of heat lost through transmission, broken down by individual thermal zones. The highest losses were observed in ground floor zones due to their proximity to the unheated basement and ground. The garage showed minimal heat loss and, in some cases, even heat gain (indicated by negative values) thanks to its proximity to heated rooms and the good insulation of the partitions. Low heat loss values were recorded on the first floor thanks to the effective insulation of the roof and attic, as well as heat gained from ground floor rooms.

**Figure 18:**
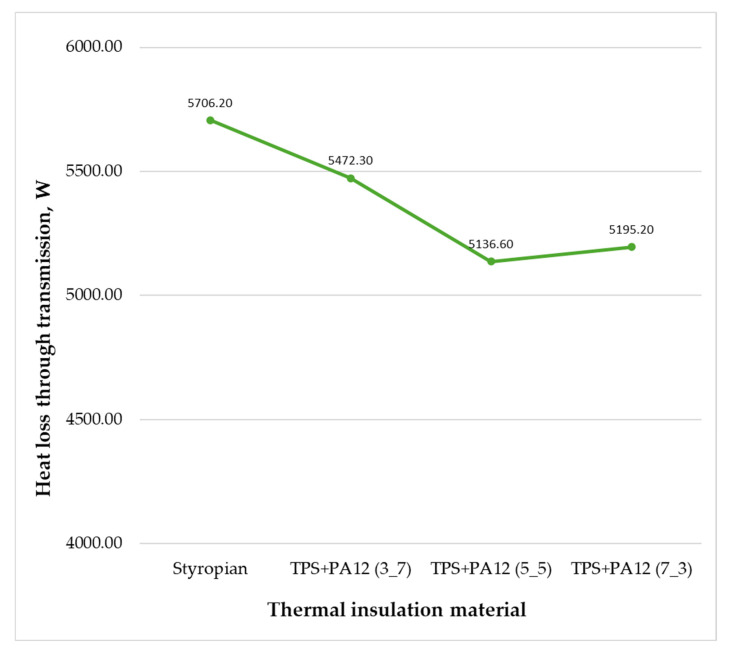
Heat loss through transmission for rooms insulated with prototype thermal insulation materials (original work).

Figure 18 illustrates the impact of different insulation materials on heat loss through transmission. In a real-world scenario, the total heat loss through transmission was 5706.2 W, and the use of each of the prototype materials reduced this amount. The most effective solution was using TPS + PA12 at a 5:5 ratio, reducing heat loss to 5136.6 W. The least effective solution was using TPS + PA12 at a 3:7 ratio, reducing heat loss to 5472.3 W.

## 5. Conclusions

The fabrication of prototype thermal insulation materials using 3D printing technology and degradable composites, the experimental determination of their thermal insulation parameters, and the analysis of their effectiveness in construction have led to the conclusions presented in this article.

All of the manufactured prototype composites exhibit the characteristics of highly efficient thermal insulation materials that can form effective thermal barriers. Their thermal insulation properties are comparable to those of conventional industry-standard materials such as polystyrene, mineral wool, and PIR/PUR foam. Furthermore, all of the fabricated open-cell materials comply with the thermal performance requirements of PN-EN ISO 9229:2020-12 [101], which stipulates a maximum thermal conductivity of 0.065 W/(m·K).

The double-layer prototype made from TPS + PA12 at a mass ratio of 50:50 with a thickness of 60 mm performs best among the 27 tested insulation materials. It achieves a thermal conductivity of λ = 0.026 W/(m·K), a thermal resistance of R = 2.360 (m^2^·K)/W, and a thermal transmittance of U = 0.424 (W/(m^2^·K)).

The most favorable mass ratio for blending thermoplastic starch (TPS) with polyamide 12 (PA12) is found to be 50:50. This proportion yields the most beneficial values for thermal conductivity, thermal transmittance, and thermal resistance, outperforming the 30:70 and 70:30 ratios. The balanced 50:50 mixture forms a structure that most effectively limits heat transfer. Excess of either TPS or PA12 has a negative impact on the material’s thermal performance compared with the balanced blend.

Analysis shows that insulation thickness is the most critical factor influencing the thermal properties of open-cell structures. Increasing the thickness always results in a lower thermal conductivity coefficient.

The number of layers also affects a material’s thermal insulation properties. Thicker open-cell composites allow air to circulate within their pores. Using multiple thinner layers mitigates this effect; each layer, separated by a spacer, acts as a barrier to air movement, thereby limiting convection and improving the system’s thermal insulation properties overall.

Replacing 12 cm thick polystyrene with prototype thermal insulation materials of the same thickness in the external walls of the single-family house built in 2009 has a positive effect on its energy performance. This reduces heat losses through transmission and the demand for usable energy for heating and ventilation, thus improving the building’s overall energy efficiency.

Using prototype thermal insulation materials with a TPS + PA12 ratio of 50:50 or 70:30 to insulate the external walls ensures compliance with the current technical requirements (WT2021), which stipulate a maximum U-value of 0.20 W/(m^2^K) for external walls in rooms where the indoor temperature is at least 16 °C.

The results of this study have prompted the authors to plan further research on the cellular composites herein analyzed. These studies will determine the mechanical properties of the composites, such as their compressive and impact strength. Additionally, the authors are investigating thermal stability using DSC/TG analyses. They also plan to assess long-term stability under real operating conditions involving temperature and humidity fluctuations. The authors also plan to evaluate the fire safety and fire resistance of the composite materials used to produce the cellular structures. Future research will focus on optimizing the thermal and mechanical properties of 3D-printed insulation materials made from renewable or biodegradable sources, such as TPS and suitable plasticizers. Optimizing the printing processes appears to be key to driving innovation in multifunctional and “smart” insulation materials.

## Figures and Tables

**Table 1:** Particle-reinforced composites (dispersion-strengthened).

Authors	Research Material	Composite Type	Application	Key Properties	Technology	Sustainability Notes
Kurańska et al. [17]	Polyurethane foams from bio-polyols (fruit seed oils)	Particle-reinforced (dispersion)	Thermal insulation	λ = 0.035–0.043 W/(m·K)	Oil transesterification, foaming	Renewable resources, seed waste
Gavrilović-Grmuša et al. [18]	Bio-epoxies based on lignin and tannic acid	Particle-reinforced (dispersion)	Wood adhesives	τt = 5.64–10.87 MPa (shear strength)	Resin chemical modification	Natural polyphenols, reduced synthetic hardeners
Mohan et al. [19]	Panels from cotton microdust + coir dust	Particle-reinforced (dispersion)	Thermal insulation	λ = 0.02–0.09 W/(m·K)	Panel forming, dust composition	Use of textile waste
Varamesh et al. [20]	Bio-aerogel (phytic acid + chitosan, cellulose)	Particle-reinforced (dispersion)	Thermal insulation	λ = 0.036–0.038 W/(m·K)	Layered assembly, crosslinking	Fully bio-based raw materials
Hilal et al. [21]	Self-compacting concrete with sunflower and walnut ash	Particle-reinforced (dispersion)	Structural (lightweight concrete)	σc = 14–33 MPa; σf = 1.9–5.2 MPa; σt = 1.1–3.0 MPa	Conventional concrete mixing	Partial cement replacement with ash, lower density
Anwajler [22]	3D-printed insulation composites (soybean oil + wastepaper ash)	Particle-reinforced (dispersion)	Thermal insulation	U = 0.016 W/m·K; LOI = 56–63%	3D printing (Voronoi, Rhino/Grasshopper)	Waste utilization, bio-based resin
Ibraheem & Bdaiwi [23]	Polyester composites with sidr leaf powder	Particle-reinforced (dispersion)	Thermal + mechanical insulation	σc = 34.5–48.7 MPa; λ = 0.101–0.190 W/(m·K)	Hand layup forming	Plant-based powder, regional waste
Cigarruista Solís et al. [24]	Insulation panels from rice husk + rice flour	Particle-reinforced (dispersion)	Thermal insulation	λ = 0.073 W/(m·K)	Mold forming, drying	Local raw materials, agro-waste
Raja et al. [25]	Epoxy with Ipomoea carnea fibers + bran filler	Particle/fiber-reinforced (dispersion)	Wall panels (antibacterial)	σt = 16.4–25.0 MPa; σf = 17.0–27.4 MPa	Hand layup	Natural fibers, hygienic properties
Aguillón et al. [26]	Boards from brewers’ spent grain + sorbitol epoxy	Particle-reinforced (dispersion)	Furniture/construction boards	σf = 33.3–51.5 MPa	Thermopressing	Brewery waste, wood-like material
Wan et al. [27]	Plasters with rice husk addition (800 μm granules)	Particle-reinforced (dispersion)	Thermal insulation	λ = 0.67–0.83 W/(m·K)	Mixing with plaster	Bio-additive in traditional plasters
Sergi et al. [28]	PLA reinforced with linoleum dust (wood flour, cork, jute)	Particle-reinforced (dispersion)	Decorative elements, lightweight panels	σt = 49–69 MPa; σf = 75–98 MPa	PLA molding, extrusion	Linoleum waste, biodegradable PLA
Fernandes et al. [29]	Glass foams with sugarcane bagasse ash	Particle-reinforced (dispersion)	Insulation, lightweight aggregate	σc = 0.48–0.58 MPa; λ = 0.05–0.07 W/(m·K)	Sintering at 750–850 °C	Bagasse ash, agro-waste, glass
Pop et al. [30]	Acoustic insulation from cellulose + beeswax, fir resin, natural fillers	Particle-reinforced (dispersion)	Acoustic insulation	SAC = 0.15–0.78; λ = 0.05–0.08 (W/m·K)	Panel forming	Fully natural materials

**Table 2:** Large-particle-reinforced composites.

Authors	Research Material	Composite Type	Application	Key Properties	Technology	Sustainability Notes
Fayzullin et al. [31]	Polypropylene composite with wood flour, rice husk, sunflower husk (enzymatically modified)	Large-particle-reinforced (0.1–1 mm)	Structural applications	σt = 21.0–31.9 MPa	Surface enzymatic modification	Improved filler–matrix bonding, renewable fillers
Jamal et al. [32]	Rice husk fiber + recycled polyethylene	Large-particle-reinforced (0.1–1 mm)	Partition panels	σt = 0.52–0.60 MPa; σf = 19.0–27.2 MPa	Blending RPE + RHF	Waste reuse, recycled PE
Grzybek et al. [33]	Pine wood particleboard with ethyl palmitate (PCM) + fire retardants	Large-particle-reinforced (0.1–1 mm)	Wall panels with PCM storage	Latent heat ≈ 50 J/g; PHRR = 348–548 kW/m^2^; THR = 82–213 MJ/m^2^	Hot pressing with additives	Thermal storage, fire safety, paper/clay waste
Bonifacio & Archbold [34]	Limestone-based composites with oat/rice husks	Large-particle-reinforced (0.1–1 mm)	Binder composites	-	Binder mixing, surface coating (linseed oil)	Delayed degradation, renewable aggregates
Buda & Pucinotti [35]	Natural hydraulic lime mortar + cork (15–30%)	Large-particle-reinforced (1–2 mm)	Mortar (binder/insulation)	σc = 2.16–3.35 MPa; σf = 2.34–3.87 MPa; λ = 0.39–0.45 W/(m·K)	Mixing, curing	Local cork, renewable resource
Dymek et al. [36]	Bio-based polyurethane foams + cork granules (from cooking oil)	Large-particle-reinforced (1–2 mm)	Foams (insulation, cushioning)	σc = 0.283–0.344 MPa; λ = 0.04–0.07 W/(m·K)	Foam forming with cork	Reused cooking oil, cork waste
Sergi et al. [37]	Hot-compressed cork planks	Large-particle-reinforced (1–2 mm)	Deck boards, interior elements	σt = 7.98–9.27 MPa; σf = 12.8–16.4 MPa; λ = 0.24–0.68 W/(m·K)	Hot compression	Agglomerated cork, renewable
Krumins et al. [38]	Bio-based particleboards (branches, needles, bark) + carbohydrate binder	Large-particle-reinforced (2–5 mm)	Boards	σf = 2.13–9.99 MPa	Hot pressing (140–160 °C)	Forest waste as reinforcement and binder
Bendaikha & Yaseri [39]	Straw-based bio-insulation (straw + aloe vera + sodium bicarbonate)	Large-particle-reinforced (2–5 mm)	Pipe insulation (geothermal)	Thermal gradient ~9 °C	Mold forming + coating	Straw waste, natural additives
Mucsi et al. [40]	Coconut coir + reed straw panels (with MDI binder)	Large-particle-reinforced (2–5 mm)	Insulation panels	λ = 0.08–0.10 W/(m·K); σf = 2.41–6.33 MPa	Hot pressing with MDI	Agro-fibers, renewable
Glenn et al. [41]	Cellulose fiber foams + paperboard reinforcements	Large-particle-reinforced (2–5 mm)	Packaging/insulation foams	λ = 0.039–0.049 W/(m·K); σf = 0.038–0.460 MPa; σc = 0.001–0.305 MPa	Foaming, starch binding	Paper waste, biodegradable
Rodríguez et al. [42]	Rice husk panels (pulping + NaOH)	Large-particle-reinforced (5–10 mm)	Thermal + acoustic insulation	λ = 0.037–0.042 W/(m·K); NRC = 0.77–0.98	Pulping, molding	Agro-waste, high acoustic absorption
Mohammed et al. [43]	Particleboards (bagasse, kenaf, cotton stalk) + casein/tannin adhesives	Large-particle-reinforced (>10 mm)	Furniture, wall panels, insulation	σf = 1.6–15.6 MPa; λ = 0.050–0.089 W/(m·K)	Hot pressing with bio-adhesives	Agro-residues, bio-adhesives
Kamalizad & Morshed [44]	Compressed earth blocks + sand-coated reed reinforcement	Large-particle-reinforced (>10 mm)	Structural blocks	σt = 40.9 MPa (reinforced); lateral displacement +76%	Manual pressing + reed reinforcement	Local earth, low-energy, seismic improvement

**Table 3:** Fiber-reinforced composites.

Authors	Research Material	Composite Type	Application	Key Properties	Technology	Sustainability Notes
Tasgin et al. [45].	Epoxy composites with cotton and sisal fibers	Continuous fiber-reinforced	Semi-structural panels	σt = 15.3–52.8 MPa; λ = 0.70–1.02 W/(m·K)	VARTM (vacuum-assisted resin transfer molding)	Natural fibers, renewable
Spyridonos et al. [46]	Pultruded hemp fiber profiles + bio-resin	Continuous fiber-reinforced	Cylindrical profiles (bending loads)	σf = 247–311 MPa; bending modulus = 21 GPa	Pultrusion	Hemp fibers, renewable
Tasgin et al. [45]	Discontinuous fiber composites (sisal, coir)	Discontinuous aligned fiber	Thermal insulation	λ = 0.187 W/(m·K) (coir); moderate tensile strength	Compression molding	Plant fibers
Han et al. [47]	Densified bamboo fiber composite (aligned)	Discontinuous aligned fiber	Structural (wood-like material)	σt = 421.5 MPa; σf = 211.1 MPa	Hot pressing with resin	High strength, renewable bamboo
Urdanpilleta et al. [48]	Soy protein + Latxa sheep wool (porous biocomposites)	Random cut fiber composite	Acoustic insulation	SAC ≈ 0.95 at 4000 Hz; λ = 0.04–0.07 W/(m·K)	Freeze-drying	Sheep wool waste, biodegradable
Segura et al. [49]	Fruit stone particles + coconut fiber panels	Random cut fiber composite	Acoustic/thermal insulation	λ = 0.145–0.159 W/(m·K); SAC = 0.7–0.95	Panel pressing	Agro-waste, natural fibers
Ali et al. [50]	Date palm fibers + pineapple leaf fibers + PVAc resin	Random cut fiber composite	Thermal/acoustic insulation	λ = 0.054–0.075 W/(m·K); SAC = 0.43–0.85	Compression molding	Agro-waste fibers
Kharshiduzzaman et al. [51]	Rattan + date palm fibers (NaOH treated)	Random cut fiber composite	Interior partitions, panels	σt = 4.6–12.5 MPa; σf = 14.3–39.1 MPa	Mold pressing	Renewable agro-fibers
Krishnasamy et al. [52]	Epoxy composites reinforced with coir/jute fibers	Random cut fiber composite	Light insulation panels	λ = 0.11–0.156 W/(m·K); SAC = 0.1–0.44	Compression molding	Natural coir/jute
Alazzawi et al. [53]	Epoxy composites with hemp, jute, date palm fibers	Random cut fiber composite	Insulation + structural panels	λ = 0.051–0.084 W/(m·K); σc = 64–70 MPa	ISO-179 cutting, resin molding	Plant fibers, renewable
Ariharasudhan et al. [54]	Bagasse + jute fiber composites with PVA	Random cut fiber composite	Load-bearing applications	σt = 6.7–7.0 MPa; σf = 12–14.6 MPa; λ = 0.112–0.156 W/(m·K)	Hand layup/compression	Agro-waste fibers
Trocinski et al. [55]	Gypsum + hemp fibers (Poland)	Random cut fiber composite	Lightweight gypsum boards	σf = 2.9–5.2 MPa; σt = 0.64–1.02 MPa	Casting with gypsum	Hemp fibers, renewable
Greco et al. [56]	Metakaolin–lime mortar + Spartium junceum fibers	Random cut fiber composite	Masonry reinforcement	σc = 6.7–12.5 MPa; σf = 0.8–2.8 MPa	Mortar mixing	Natural textile fibers
Jové-Sandoval et al. [57].	Adobe clay + wheat straw/sawdust fibers	Random cut fiber composite	Thermal insulation panels	λ = 0.05–0.15 W/(m·K)	Mixing clay slurry	Agro-waste fibers, local
Jadhav et al. [58]	Hemp fiber + silica xerogel composites	Random cut fiber composite	Thermal insulation (fire retardant)	λ = 0.031–0.036 W/(m·K)	Xerogel preparation, pressing	Recycled hemp fibers
Kabore & Ouellet-Plamondon [59]	Cob (clay + fibers) samples	Random cut fiber composite	Non-load-bearing, insulating filler	σc = 1.8–4.6 MPa; λ = 0.2–0.5 W/(m·K)	Handcrafted cob drying	Local clay, plant fibers
Kebede et al. [60]	Polyester composites with water lily fibers	Random cut fiber composite	Structural applications	σt = 43.8–95.7 MPa; σf = 57.9–110.7 MPa	Polyester resin pressing	Invasive aquatic plants reused

**Table 4:** Layer-reinforced composites.

Authors	Research Material	Composite Type	Application	Key Properties	Technology	Sustainability Notes
Bąk et al. [61]	Multilayer geopolymer composites with coconut/jute/hemp/flax felt/wool	Layer-reinforced (laminates)	Building envelopes (insulation panels)	λ = 0.805–1.177 W/(m·K)	Lamination + fiberglass reinforcement	Natural insulating mats, renewable
Varma et al. [62]	Concrete cylinders wrapped with jute + basalt fibers	Layer-reinforced (laminates)	Structural strengthening (columns)	σf = 73.6–110.7 MPa (flexural strength); compressive axial stress = 40.2 MPa	Hand layup wrapping	Basalt + jute natural fibers
Abu-Saleem & Gattas [63]	Timber–cardboard sandwich columns (plywood + waste cardboard)	Layer-reinforced (sandwich)	Lightweight structural columns	σc = 23.4–25.4 MPa; ultimate load = 34–84 kN	Sandwich panel assembly	Recycled cardboard core, plywood facings
Abu-Saleem & Gattas [64]	Timber–cardboard sandwich beams	Layer-reinforced (sandwich)	Lightweight beams	σf = 26.7–28.6 MPa; ultimate load ≈ 13 kN	Sandwich beam assembly	Recycled cardboard, plywood skins

**Table 5:** Summary of studies on biomaterials for 3D printing.

Material/System	Three-Dimensional Printing Technique	Key Results/Observations	Source
PLA/TPS	FDM	Classical two-step approach; compatibility issues, improved properties with additives.	Li & Huneault [77]
PLA + 60% TPS	FDM	Elongation at break increased by 77%; greater ductility but lower stiffness.	Souri Rudabadi et al. [78]
PLA/TPS bio-based filament	FDM	Successful printing of porous structures and anatomical models; high printability.	Haryńska et al. [79]
PLA/TPS/PBAT	FDM	Low-cost filaments; stable and repeatable printing, good mechanical properties.	Ju et al. [80]
PLA/TPS + pyrogallol	Extrusion (no 3D printing)	One-step strategy; improved phase compatibility; no 3D printing test.	Qin et al. [81]
PLA/PBS	FDM	Elongation increased by 150–300% with compatibilisers; ↑ costs.	Cai et al. [82]
TPS z hemp shives	-	Plasticization of starch with hemp shives and glycerol; a description of methods and the properties of TPS (thermoplastic starch) as a base for composites.	Foret et al. [83]
PLA/PBAT	FDM	Flexible composites; improved deformability, compatibilizers required.	Miao et al. [84]
PLA + elastomers (NR/PU)	FDM	Impact strength ↑ 2–5×; reduced biodegradability.	Hamidi et al. [85,86]
PCL	SLS	Tissue/bone scaffolds, controlled porosity and geometry; good cell colonization.	Williams et al. [87]
PCL/HA	SLS	Bioactive bone composites. Porous 70/30% structures; bioactivity; modulus 0.6–2.3 MPa; σᵧ 0.1–0.6 MPa; effect of laser power and orientation.	Wiria et al. [88]; Eosoly et al. [89]
PLLA/PLGA + HA, β-TCP	SLS	Resorbable scaffolds. SLS-sintered composites; suitable sintering window; potential as bone substitute.	Simpson et al. [90]
PEEK/HA	SLS	Load-bearing implants; bioactivity; mechanical properties tailored for orthopedic applications.	Rodzen et al. [91]
PLA (modified)	-	Biocomposites. Narrow SLS window; modifications (e.g., nanoclay, wood fibers) improve powder stability and processability.	Hao, Savalani et al. [92]
PVA/HA	-	Bioactive composites. Sintered PVA/HA powders produce porous bioactive structures; quality dependent on powder morphology.	Wiria et al. [93]

**Table 6:** Geometry of the prototype of the designed thermal insulation material (own elaboration).

No.	A, mm	d, mm	n, -
1	50	20	1
2	50	20	2
3	50	20	3
4	50	40	1
5	50	40	2
6	50	40	3
7	50	60	1
8	50	60	2
9	50	60	3

**Table 7:** Variants of mass concentrations of raw materials for 3D-printed prototype thermal insulation materials (original design).

Variant	Material	Percent by Weight Concentration [wt.%]
1	PA12 + TPS	70:30
2	PA12 + TPS	50:50
3	PA12 + TPS	30:70

**Table 8:** The accuracy of the measuring instruments.

Measuring Device	Accuracy
K-type thermocouple (HELUKABEL Polska Sp. z o.o., Radziejowice, Poland)	0.1 K
FHF04SC heat flux sensor	11 μV/(W/m^2^)
Vernier caliper (GEKO SPÓŁKA Z OGRANICZNĄ ODPOWIEDZIALNOŚCIA SPÓŁKA KOMANDYTOWA, Kietlin, Poland)	0.05 mm

**Table 9:** One-way significance tests for λ, W/mK.

Symbol That Identifies the Input Factors	SS	Degrees of Freedom	MS	F	*p*
Absolute term	0.117051	1	0.117051	251351.7	0.00
%_PA12	0.000084	2	0.000042	90.4	0.00
d	0.003446	2	0.001723	3700.4	0.00
n	0.000217	2	0.000108	232.8	0.00
%_PA12*d	0.000114	4	0.000028	61.0	0.00
%_PA12*n	0.000014	4	0.000004	7.7	0.00
d*n	0.000160	4	0.000040	85.9	0.00
%_PA12*d*n	0.000044	8	0.000006	11.8	0.00
Error	0.000025	54	0.000000		

**Table 10:** The results of the measurements of the mean thermal conductivity (λ), thermal resistance (R), and thermal transmittance (U) for prototype thermal insulation materials with a layer count of n and a thickness of d, fabricated from TPS + PA12 in a 3:7 mass ratio, including measurement uncertainty, are presented in this work.

d	20 mm			40 mm			60 mm		
n. -	1	2	3	1	2	3	1	2	3
λmean. W/mK	0.049	0.044	0.038	0.044	0.041	0.041	0.032	0.032	0.033
u (λmean)	0.0022	0.0022	0.0020	0.0028	0.0027	0.0027	0.0023	0.0022	0.0023
Rmean. m^2^K/W	0.406	0.456	0.530	0.927	0.964	0.983	1.867	1.864	1.861
u (Rmean)	0.0180	0.0224	0.0271	0.0596	0.0627	0.0645	0.1330	0.1277	0.1289
Umean. W/m^2^K	2.461	2.195	1.889	1.079	1.037	1.017	0.536	0.537	0.537
u (U Umean)	0.1089	0.1077	0.0965	0.0694	0.0674	0.0667	0.0382	0.0368	0.0372

**Table 11:** The results of the measurements of the mean thermal conductivity (λ), thermal resistance (R), and thermal transmittance (U) for prototype thermal insulation materials with a layer count of n and a thickness of d, fabricated from TPS + PA12 in a 5:5 mass ratio, including measurement uncertainty, are presented in this work.

d	20 mm			40 mm			60 mm		
n. -	1	2	3	1	2	3	1	2	3
λmean. W/mK	0.046	0.044	0.041	0.045	0.038	0.040	0.027	0.026	0.026
u (λmean)	0.0022	0.0022	0.0020	0.0028	0.0027	0.0027	0.0023	0.0022	0.0023
Rmean. m^2^K/W	0.433	0.457	0.489	0.890	1.054	1.007	2.197	2.360	2.278
u (Rmean)	0.0204	0.0224	0.0232	0.0561	0.0748	0.0684	0.1815	0.2059	0.1967
Umean. W/m^2^K	2.311	2.188	2.046	1.123	0.948	0.993	0.455	0.424	0.439
u (U Umean)	0.1090	0.1074	0.0973	0.0708	0.0673	0.0674	0.0376	0.0370	0.0379

**Table 12:** The results of the measurements of the mean thermal conductivity (λ), thermal resistance (R), and thermal transmittance (U) for prototype thermal insulation materials with a layer count of n and a thickness of d, fabricated from TPS + PA12 in a 7:3 mass ratio, including measurement uncertainty, are presented in this work.

d	20 mm			40 mm			60 mm		
n. -	1	2	3	1	2	3	1	2y	3
λmean. W/mK	0.049	0.045	0.041	0.041	0.039	0.038	0.028	0.028	0.028
u (λmean)	0.0023	0.0026	0.0029	0.0027	0.0028	0.0025	0.0019	0.0021	0.0021
Rmean. m^2^K/W	0.413	0.450	0.493	0.971	1.021	1.048	2.152	2.125	2.136
u (Rmean)	0.0193	0.0258	0.0348	0.0640	0.0712	0.0678	0.1488	0.1557	0.1571
Umean. W/m^2^K	2.420	2.224	2.030	1.030	0.979	0.955	0.465	0.471	0.468
u (U Umean)	0.1129	0.1275	0.1435	0.0679	0.0683	0.0618	0.0321	0.0345	0.0344

**Table 13:** Variants of 12 cm thick prototype thermal insulation materials made from TPS + PA12 at different mass ratios (own work).

Material	Trendline Function	Thermal Conductivity Coefficient (λ) for a Thickness of 12 cm (Three-Layer)
Polystyrene	Not applicable	0.0400 $\frac{W}{m\cdot K}$
TPS + PA12 (30:70)	$y=0.0559*x^{-0.114}$	0.0324 $\frac{W}{m\cdot K}$
TPS + PA12 (50:50)	$y=0.1299*x^{-0.364}$	0.0227 $\frac{W}{m\cdot K}$
TPS + PA12 (70:30)	$y=0.1096*x^{-0.315}$	0.0243 $\frac{W}{m\cdot K}$

**Table 14:** Comparison of the thermal parameters of a single-family building with external wall insulation and various types of insulation material (original work).

Building Parameter	Jednostka	Styropian, Λ = 0.0400	TPS + PA12 (30:70), Λ = 0.0324	TPS + PA12 (50:50), Λ = 0.0227	TPS + PA12 (70:30), Λ = 0.0243
Design heat loss through transmission, ΦT	kW	5.43	5.19	4.86	4.92
Heat loss through external walls, Q	kW	1.57	1.34	1.00	1.06
Design ventilation heat loss, ΦV	kW	3.52	3.52	3.52	3.52
Total design heat load, ΦHL	kW	8.95	8.72	8.38	8.44
Design heat load per surface area, ΦA	W/m^2^	57.09	55.60	53.45	53.83
Total heat loss through transmission, QH,tr	kWh/rok	14,190.19	13,557.97	12,650.75	12,809.01
Total heat loss through ventilation, QH,ve	kWh/rok	7051.84	7051.84	7051.84	7051.84
Annual useful heat demand for heating and ventilation, QH,nd	kWh/rok	10,989.56	10,540.79	9902.41	10,013.27
Annual energy demand index for heating and ventilation, EU	kWh/m^2^rok	70.09	67.22	63.15	63.86

**Table 15:** Heat demand of rooms insulated with prototype thermal insulation materials (own elaboration).

Thermal Insulation Material d = 12 cm		Styropian, Λ = 0.0400	TPS + PA12 (30:70), Λ = 0.0324	TPS + PA12 (50:50), Λ = 0.0227	TPS + PA12 (70:30), Λ = 0.0243
Zone name	A, m^2^	Heat demand for heating and ventilation, kWh/rok			
Garage (ground floor) 5 °C	24.80	0.00	0.00	0.00	0.00
Living rooms (ground floor) 20 °C	59.90	3914.53	3728.32	3463.82	3509.72
Bathroom (ground floor) 24 °C	7.80	1639.06	1587.51	1513.84	1526.66
Living rooms (first floor) 20 °C	55.10	3961.04	3779.42	3521.21	3566.04
Bathroom (first floor I) 24 °C	9.20	1474.93	1445.55	1403.53	1410.85
Total	156.80	10,989.56	10,540.80	9902.40	10,013.27

**Table 16:** Heat loss through transmission for rooms insulated with prototype thermal insulation materials (own elaboration).

Thermal Insulation Material d = 12 cm		Styropian, Λ = 0.0400	TPS + PA12 (30:70), Λ = 0.0324	TPS + PA12 (50:50), Λ = 0.0227	TPS + PA12 (70:30), Λ = 0.0243
Zone name	A, m^2^	Heat loss through transmission, ΦTi, W			
Garage (ground floor) 5 °C	24.80	30.30	1.00	−41.10	−33.70
Living rooms (ground floor) 20 °C	59.90	2269.10	2178.40	2048.30	2071.00
Bathroom (ground floor) 24 °C	7.80	2227.60	2141.40	2017.60	2039.20
Living rooms (first floor) 20 °C	55.10	601.30	583.90	559.00	563.30
Bathroom (first floor I) 24 °C	9.20	577.90	567.60	552.80	555.40
Total	156.80	5706.20	5472.30	5136.60	5195.20

## Data Availability

The original contributions presented in this study are included in the article. Further inquiries can be directed to the corresponding author.
